# Unique Changes in the Lung Microbiome following the Development of Chronic Lung Allograft Dysfunction

**DOI:** 10.3390/microorganisms12020287

**Published:** 2024-01-29

**Authors:** Yeuni Yu, Yun Hak Kim, Woo Hyun Cho, Dohyung Kim, Min Wook So, Bong Soo Son, Hye Ju Yeo

**Affiliations:** 1 Biomedical Research Institute, School of Medicine, Pusan National University, Yangsan 50612, Republic of Korea; yuyeuni0528@gmail.com; 2 Department of Anatomy and Department of Biomedical Informatics, School of Medicine, Pusan National University, Yangsan 50612, Republic of Korea; yunhak10510@pusan.ac.kr; 3 Division of Pulmonary, Allergy and Critical Care Medicine, Department of Internal Medicine, Pusan National University Yangsan Hospital, Pusan National University School of Medicine, Yangsan 50612, Republic of Korea; popeyes0212@hanmail.net; 4 Department of Thoracic and Cardiovascular Surgery, Pusan National University Yangsan Hospital, Pusan National University School of Medicine, Yangsan 50612, Republic of Korea; yumccs@nate.com; 5 Division of Rheumatology, Department of Internal Medicine, Pusan National University Yangsan Hospital, Pusan National University School of Medicine, Yangsan 50612, Republic of Korea; thalsdnrso@naver.com; 6 Research Institute for Convergence of Biomedical Science and Technology, Pusan National University Yangsan Hospital, Pusan National University School of Medicine, Yangsan 50612, Republic of Korea

**Keywords:** CLAD, diversity, Klebsiella, lung microbiome, lung transplant

## Abstract

The importance of lung microbiome changes in developing chronic lung allograft dysfunction (CLAD) after lung transplantation is poorly understood. The lung microbiome–immune interaction may be critical in developing CLAD. In this context, examining alterations in the microbiome and immune cells of the lungs following CLAD, in comparison to the lung condition immediately after transplantation, can offer valuable insights. Four adult patients who underwent lung retransplantation between January 2019 and June 2020 were included in this study. Lung tissues were collected from the same four individuals at two different time points: at the time of the first transplant and at the time of the explantation of CLAD lungs at retransplantation due to CLAD. We analyzed whole-genome sequencing using the Kraken2 algorithm and quantified the cell fractionation from the bulk tissue gene expression profile for each lung tissue. Finally, we compared the differences in lung microbiome and immune cells between the lung tissues of these two time points. The median age of the recipients was 57 years, and most (75%) had undergone lung transplants for idiopathic pulmonary fibrosis. All patients were administered basiliximab for induction therapy and were maintained on three immunosuppressants. The median CLAD-free survival term was 693.5 days, and the median time to redo the lung transplant was 843.5 days. Bacterial diversity was significantly lower in the CLAD lungs than at transplantation. Bacterial diversity tended to decrease according to the severity of the CLAD. *Aerococcus*, *Caldiericum*, *Croceibacter*, *Leptolyngbya*, and *Pulveribacter* genera were uniquely identified in CLAD, whereas no taxa were identified in lungs at transplantation. In particular, six taxa, including *Croceibacter atlanticus*, *Caldiserium exile*, *Dolichospermum compactum*, *Stappia sp. ES.058*, *Kinetoplastibacterium sorsogonicusi*, and *Pulveribacter suum* were uniquely detected in CLAD. Among immune cells, CD8+ T cells were significantly increased, while neutrophils were decreased in the CLAD lung. In conclusion, unique changes in lung microbiome and immune cell composition were confirmed in lung tissue after CLAD compared to at transplantation.

## 1. Introduction

The morbidity and mortality of lung transplantation has improved over the past three decades. In recent years, the International Society for Heart and Lung Transplantation (ISHLT) has reported an increase in the 1-year survival rates of lung transplantation, reaching 90% [1]. Despite the improvement in early mortality, the rate of decline in lung function after 1 year has not changed substantially; moreover, the long-term survival is still lower than that of other solid organ transplants. Chronic lung allograft dysfunction (CLAD) is the primary limiting factor for long-term survival after lung transplantation [2]. Several factors specific to the lungs, including gastroesophageal reflux and recurrent infection, contribute to CLAD [3,4].

With the introduction of high-throughput sequencing methods, our understanding of the human lung microbiome has expanded significantly [5]. There are several studies on the relationship between inflammation and changes in the lung microbiome, and the development of chronic lung disease [6]. The lung microbiome of lung transplant patients has also been found to be unique compared to that of non-transplant patients [7]. It has also been suggested that changes in the microbiome of the bronchoalveolar lavage (BAL) fluid after transplantation are associated with CLAD development [8]. The lung microbiome near CLAD onset shows similar characteristics, regardless of the CLAD phenotype [9]; thus, the microbiome of transplanted lungs may play a critical role in CLAD onset by interacting with the recipient’s innate and adaptive immune systems [10,11]. However, the mechanisms linking the lung microbiome to immune system interactions in CLAD are still poorly delineated. It is unclear which individual bacterial taxa are definitively associated with CLAD development or mortality [7]. In this context, an evaluation of the changes in the microbiome and immune cells of the lungs after CLAD, compared to the lung condition immediately after transplantation, can provide important insights. A recent report has shown that the deconvolution of gene expression data using cell type identification by relative subset estimation of RNA transcriptome (CIBERSORT) can yield valuable insights into the composition of immune cells [12,13]. We investigated differences in the lung microbiome and immune cells between lung tissue at transplantation and lung tissue in which CLAD developed in a cohort of four patients who underwent retransplantation due to CLAD.

## 2. Materials and Methods

### 2.1. DNA Sampling

Four adult patients with tissue sampled at different time intervals who underwent lung retransplantation between January 2019 and June 2020 were included in this study. Their tissue was obtained from the Pusan National University Yangsan Hospital (PNUYH) Biobank. All reviewed tissue from the biobank is anonymized, and the need for informed consent was waived. Lung tissue at the time of transplantation is defined as a remnant of an oversized donor lung preserved in our biobank. CLAD lung tissue comprised explanted lung tissue at the time of redo transplantation. CLAD was diagnosed based on ISHLT guidelines [2]. We received anonymized, well-preserved samples and clinical data from the biobank. The biobank’s standard tissue conservation technique is presented in Supplementary Method S1. The Institutional Review Board of PNUYH approved this retrospective cohort study conducted at a single center (05-2021-163). We performed whole genome and RNA sequencing for each lung tissue sample. We analyzed the differences in lung microbiome characteristics and immune cells between lung tissue at transplantation and CLAD lung tissue. A sufficient amount of lung tissue was used for analysis to obtain the appropriate lung biomass. To avoid potential contamination, we resected and stored lung tissue under sterile surgical conditions for DNA and RNA extraction and library preparation. There was no potential contamination by background or external DNA during the operating procedures and sequencing library preparation. Sufficient bacterial biomass was identified in the resected lung tissue.

### 2.2. DNA Extraction, Library Construction, and Sequencing

DNA extraction was performed using the Maxwell^®^ 16 Formalin-Fixed, Paraffin-Embedded (FFPE) Plus LEV DNA Purification Kit (Promega Corp., Madison, WI, USA) in a final volume of 300 µL, according to the manufacturer’s instructions. Details of DNA extraction, library construction, and sequencing are provided in Supplementary Methods S2.

### 2.3. RNA Extraction, Library Construction, and Sequencing

Trizol, SDS, and RNeasy Mini Kit (Qiagen, Hilden, Germany) were used for RNA isolation and purification, with the minor modification of being applied in parallel for the extraction of RNA from FFPE tissues. The protocol included deparaffinization, proteinase K digestion, extraction, elution or hydration procedures, and DNase treatment to obtain DNase-free RNA. According to the manufacturer’s protocol, the Agilent SureSelectXT RNA Direct Kit (Agilent Technologies, Santa Clara, CA, USA) was used to construct the sequencing libraries using 100 ng of RNA. RNA extraction, library construction, and sequencing details are in Supplementary Method S3.

### 2.4. Kraken DNA Seq Microbial-Detection Pipeline

Paired-end reads were aligned to the GRCh38 reference using the Burrows-Wheeler Aligner. Sequencing reads that could not be aligned with known human reference genomes were mapped against a comprehensive database of known bacterial, archaeal, and viral microbial genomes using the ultrafast Kraken2 algorithm. The specific microbial database used in this analysis was the miniKraken DB_8GB [14] (Supplementary Figure S1). The Kraken algorithm breaks each sequencing read into k-mers and exactly matches each k-mer against a database of microbial k-mers. The set of exact k-mer matches for a given read, in turn, provides a putative taxonomy assignment of the lowest common ancestor for that read, most accurately to the genus level, to which we summarized our data. The matching and classification operations are orders of magnitude faster than performing direct genome alignments [15]. Kraken2 reports were combined into taxonomic read-abundance tables with Bracken. Newly estimated taxa counts through the Bayesian model were used in the analysis. Taxa with less than 10 counts, the default of the Bracken analysis, were removed. The proportion of microbes in the samples are represented in Supplementary Table S1.

### 2.5. CIBERSORT and Assessment of Tumor-Infiltrating Immune Cells

CIBERSORT [16] is a versatile computational method used to quantify the proportions of different cell types within bulk tissue gene expression profiles. It provides a powerful tool for analyzing and understanding the composition of cell populations in complex biological samples. Combining support vector regression with prior knowledge of expression profiles forms purified leukocyte subsets. CIBERSORT can accurately estimate the immune composition of a tissue. FPKM (fragments per kilobase of transcript per million mapped reads) values were used due to their superiority with respect to deconvolution analysis. The gene expression file was uploaded to CIBERSORT as a mixture file. CIBERSORT was executed with the following options: simultaneous use of relative and absolute modes, 100 permutations, and quantile normalization disabled. These settings allow for comprehensive analysis and accurate estimation of cell fractions within the sample. CIBERSORT transformed the expression of genes into the levels of immune cells by analyzing the composition and proportion of 22 tumor-infiltrating immune cells in tissue samples.

### 2.6. Statistical Analysis

All statistical analyses were performed using SPSS 26.0 version software (IBM Corp., Armonk, NY, USA) or MedCalc Statistical Software (version 18; MedCalc Software Bvba, Ostend, Belgium). Numerical results are expressed as the median (interquartile range). Continuous variables were compared using independent *t*-tests (parametric values) or the Mann–Whitney U-test (non-parametric values), as appropriate. The categorical variables were compared using the chi-square or Fisher’s exact tests. In all analyses, *p*-values < 0.05 were considered statistically significant. Abundance tables of bacteria were used to calculate diversity at the species level in R (version 4.0.3, R Foundation for Statistical Computing, Vienna, Austria) via the vegan library. Aldex2 [17] and LefSe [18] were applied to identify group-specific bacteria. In Aldex2, the *p*-value of each test was adjusted to the false discovery rate (FDR) using the Benjamini–Hochberg algorithm, in which an FDR threshold lower than 0.05 was applied to determine the significance.

## 3. Results

### 3.1. Patients’ Characteristics

The baseline patient characteristics are shown in Table 1. The median age was 57 years, and three patients underwent lung transplantation for idiopathic pulmonary fibrosis. All patients were administered basiliximab for induction of immunosuppression and were maintained on three immunosuppressants. Tacrolimus and steroids were administered to all patients. One patient received azathioprine instead of mycophenolate. All patients experienced pneumonia during the transplantation period. The median CLAD-free survival term was 693.5 days, and the median time to redo the lung transplant was 843.5 days.

### 3.2. Diversity Difference between Lungs at Transplantation and CLAD Lungs

**Figure 1:**
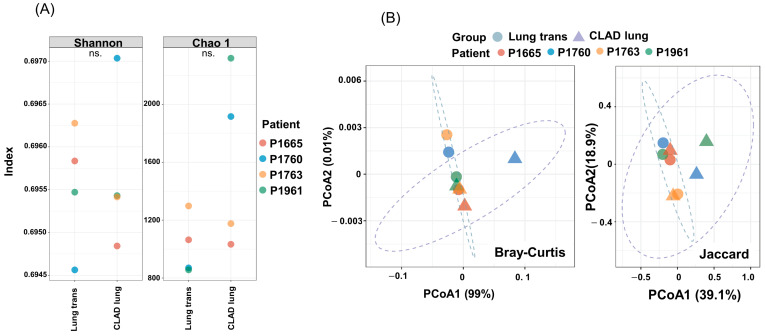
Alpha and beta diversity differences in the lung microbiome in the lung at transplantation and CLAD lung. CLAD, chronic lung allograft dysfunction. (**A**) Alpha diversity-evenness (Shannon diversity index) and alpha diversity-richness (abundance-based coverage estimator). ns.; not significant. (**B**) Principal coordinate analysis plots based on Bray–Curtis dissimilarity and the Jaccard distance in the lung at transplantation and CLAD lung (Adonis, *p* = 0.061, *p* = 0.053).

Alpha diversity was estimated using the Shannon diversity index, which relates both sample richness and evenness to the total number of observed species. The change in alpha diversity between lungs at transplantation and CLAD lungs differed for each sample. In P1760, alpha-diversity increased in both richness (chao1) and evenness (Shannon), and in P1961, the Shannon index decreased, but Chao 1 increased significantly. P1655 and P1763 showed decreased diversity for both indices (Figure 1A). Beta diversity analysis was performed using Bray–Curtis dissimilarity and the Jaccard distance, and a subsequent principal coordinate analysis (PcoA) was performed. Two-dimensional PcoA could distinctly separate the microbiota from the individual lungs at transplantation and CLAD lungs (Figure 1B). These results thus demonstrate the ability to resolve changes in bacterial diversity in the CLAD lung compared with the lung at transplantation. In patients (P1665, P1763, P1961) with a history of respiratory bacterial infection, the analysis revealed that bacterial diversity was significantly lower in the CLAD lungs compared to the lungs at transplantation. This finding suggests a potential association between reduced bacterial diversity and the development of CLAD in these patients.

### 3.3. Lung Microbiome Composition in Lungs at Transplantation and CLAD Lungs

**Figure 2:**
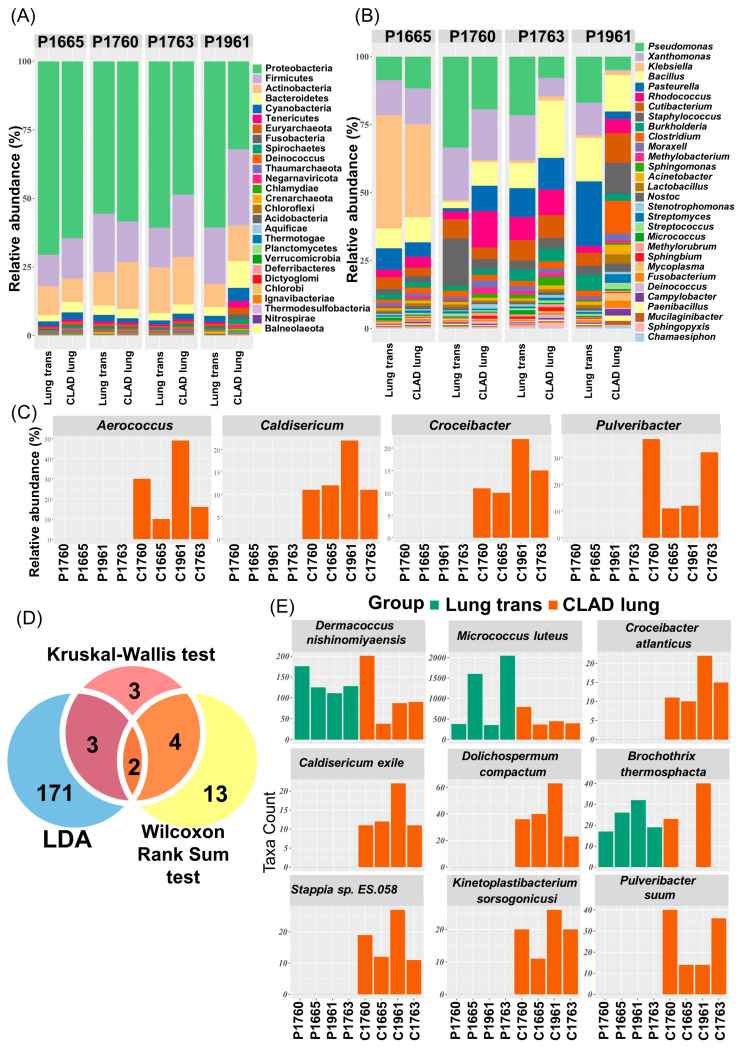
Comparison of the lung microbiota structure and abundance between the lung at transplantation and the CLAD lung. Profiling of bacterial taxa at the (**A**) phylum and (**B**) genus levels. (**C**) Genera that were identified only in CLAD. (**D**) Venn diagrams representing the consistency of differentially abundant species identified using LDA, the Aldex2-Kruskal–Wallis test, and the Aldex2-Wilcoxon rank-sum test. (**E**) The abundance of species with statistically significant differences between the lung at transplantation and CLAD lung. LDA, linear discriminant analysis.

At the phylum level, actinobacteria, firmicutes, and proteobacteria were highly abundant, accounting for approximately 75% of the total bacterial community (Figure 2A). At the genus level, the top 30 strains found in all samples with high abundance were selected and shown using a stacked bar chart (Figure 2B). Although there was a difference depending on the sample, *Pseudomonas*, *Xanthomonas*, *Bacillus*, *Pasteurella*, and *Rhodococcus* accounted for a large proportion. P1665 had more *Klebsiella* strains than the other strains, which was still high after CLAD. In most samples, the top 30 strains accounted for 70% of the total genera; however, in P1961, they only accounted for approximately 40%. This result indicates that there were many strains in small amounts. *Aerococcus*, *Caldiericum*, *Croceibacter*, *Leptolyngbya*, and *Pulveribacter* were uniquely identified in CLAD lungs, whereas no taxa were uniquely recognized in lungs at transplantation (Figure 2C). To screen for species-level taxa with significant differences between lungs at transplantation and CLAD lungs, we used Aldex2 and linear discriminant analysis (LDA). We selected taxa that were significant in at least two of the three analysis methods (Figure 2D), identified nine taxa, and confirmed their abundance, of which six taxa (*Croceibacter atlanticus*, *Caldisericum exile*, *Dolichospermum compactum*, *Stappia sp. ES.058*, *Kinetoplastibacterium sorsogonicusi*, and *Pulveribacter suum*) were identified uniquely in CLAD (Figure 2E).

### 3.4. Comparison of Estimated Immune Cells

**Figure 3:**
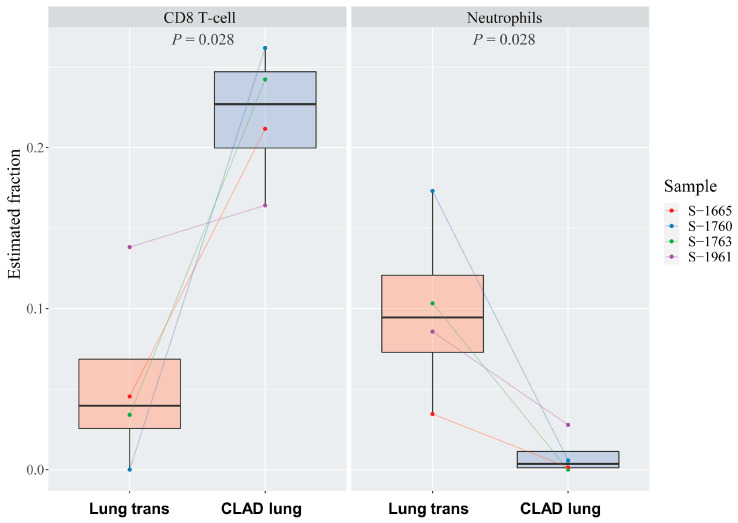
Difference in immune infiltration between the lung at transplantation and CLAD lung. Box plot of the distribution of the CIBERSORT estimated value for CD8+ T cells and neutrophils.

The gene expression data obtained from the lung at transplantation and CLAD lung tissue samples were utilized to determine the extent of immune cell infiltration using the CIBERSORT tool. CIBERSORT utilizes pre-defined gene signatures for various immune cell types, including B cell subtypes, plasma cells, T cell subtypes, natural killer cells, monocytes, macrophage subtypes, dendritic cell subtypes, mast cell subtypes, eosinophils, and neutrophils. Out of the 22 immune cell types predicted by CIBERSORT, the levels of CD8+ T cells and neutrophils exhibited a statistically significant difference between the lung at transplantation and CLAD lung (*p* < 0.05). Specifically, CD8+ T cells were found to be increased, while neutrophil levels were observed to be decreased in the CLAD condition (as depicted in Figure 3). This suggests an altered immune cell composition associated with the progression from the lung at transplantation to the CLAD lung.

## 4. Discussion

In this study, significant changes were observed in the lung microbiome and immune cells between the lung at transplantation and CLAD states in the same patients. Bacterial diversity was lower in the CLAD lungs than in lungs at the time of transplant, and bacterial diversity tended to decrease according to the severity of CLAD. In particular, in patients with a history of recurrent respiratory bacterial infections, the bacterial diversity was significantly reduced. *Croceibacter atlanticus*, *Caldisericum exile*, *Dolichospermum compactum*, *Stappia sp. ES.058*, *Kinetoplastibacterium sorsogonicusi*, and *Pulveribacter suum* were significantly higher in the CLAD lungs than in the lungs at transplantation. In terms of immune cells, CD8+ T cells were increased in CLAD. Compared with the lung at transplantation in the same patient, the CLAD lung showed a unique microbiome signature and immune cell changes.

The lung microbiome has not yet been thoroughly studied and is a potential risk factor for CLAD development. Lung transplant recipients have several unique factors affecting the balance between bacterial migration and elimination, collectively shaping the lung microbiome. Immunosuppressive medications decrease baseline immune surveillance, prophylactic antibiotics exert exogenous selection pressures, increased reflux rates lead to increased immigration by particular taxa, and surgical anastomoses and denervation result in reduced bacterial clearance [19]. Consequentially, compared with healthy individuals, lung transplant recipients may have altered respiratory microbiome characteristics, including increased bacterial burden and altered lung communities. However, the clinical significance of these differences in lung microbiota on outcomes after lung transplantation is, to date, undetermined [20]. In a previous study, the microbiome of BAL in lung transplant patients differed from that of healthy patients in terms of both bacterial load and composition [21]. In asymptomatic transplant patients, an increased bacterial burden of BAL 1 year after lung transplantation was associated with chronic rejection and mortality [7]. Currently, studies on the effects of the microbiome on CLAD development in the same patient are limited. Only one study has investigated sequential changes in the lung microbiome during the development of CLAD [8]. This study is the first to sequentially analyze the lung microbiome at the time of transplantation and the lung microbiome at CLAD in the same patient.

Currently, infection is considered a risk factor for the development of CLAD, and both acute infection and chronic lung allograft colonization with microorganisms may in-crease the risk for CLAD [22]. Infections cause a bacterial imbalance in the lung, which may result in changes in the microbial community of transplanted lungs. In particular, the bacteria *Pseudomonas aeruginosa* infection has been reported to be associated with the development of CLAD [23]. In this study, all patients experienced pneumonia at least once during the transplant period, and no bacteria were identified in the traditional culture at the time of CLAD lung removal (Table 1). However, *Proteobacteria* were the most abundant in both lungs at transplantation and CLAD lungs, and *Pseudomonas* accounted for a high proportion of the lung microbiome. Although the pathogenesis has not been fully clarified, chronic *P. aeruginosa* colonization or infection leads to persistent inflammation, causing epithelium damage [24]. This may consequently lead to the release of pro-inflammatory cytokines and epithelial alarmins, followed by immune system stimulation and pro-inflammatory fibroblast activation [25]. It has also been reported that *Pseudomonas* induces an epithelial-to-mesenchymal transition (EMT) in the bronchial epithelial cells of CLAD patients [26]. *P. aeruginosa*, via the activation of monocytic cells, may accentuate TGF-β1-driven EMT [27]. Given the well-established correlation between the lung microbiota and lung immune tone, lung microbiomes, such as *Pseudomonas,* may be a plausible key driver of post-transplant lung inflammation and allograft dysfunction independently of acute infections.

Furthermore, six taxa were uniquely identified in CLAD, all of which were rare biospheres. To date, the role of these rare species in CLAD is unclear. Despite the low abundance of rare biospheres, recent studies suggest that rare microbial taxa may be more relevant to human disease than previously expected [28]. An estimated 1.5–28% of all microbes are ‘conditionally rare taxa’, i.e., rare in most conditions but may become dominant under certain situations [29,30,31]. In particular, *Dolichospermum compactum* is one of the most ubiquitous bloom-forming cyanobacterial species. Lipopolysaccharide of cyanobacteria contributes to lung inflammation by increasing factors that promote lung inflammation and neutrophil recruitment, leading to steroid refractory airway constriction [32]. Recurrent exposure to such inflammation in lung transplant patients can lead to aberrant remodeling by bronchial epithelial damage, stimulating the immune system and activating cytokine secretion. Although this result cannot clearly show the role of rare biospheres in CLAD, the rare microbes may be essential players of host-associated microbiomes in lung transplant recipients by preventing pathogen establishment and stimulating host immunity; however, further research is required.

In this study, CD8+ T cells increased in CLAD, whereas neutrophils decreased. Compared to CD4+ T cells, CD8+ T cells are less affected by most immunosuppressive therapies that are used to prevent rejection [33]. Therefore, CD8+ T cells are considered an important barrier to the long-term survival of organ allografts [34,35]. A previous study showed a strong positive relationship between *Pasteurella* and the infiltration of CD8+ T cells in patients with lung cancer [36]. However, in this study, we were unable to confirm the relationship between specific bacteria and CD8+ T cells due to the small number of patients. Further research is required to determine whether these changes in immune cells are related to changes in the microbiome.

Our study has some limitations that warrant further investigation. First, there was a risk of selection bias, due to the small number of patients in a single center. Second, whole genome sequencing analysis using Kraken cannot provide species-level discrimination or detect virulence factors. In the future, utilizing diverse assemblers such as Diamond or MEGAN may be helpful in comprehensive microbial analysis. However, this is the first study to compare the lung microbiota in the donor lung at transplantation with the lung microbiome in the post-CLAD state in the same patient. We used human whole genome sequencing data generated from the DNBSEQ-T7 platform. Whole genome sequencing using DNBBSEQ-T7 is comparable to conventional short-read sequencing methods in terms of the error rate/quality. Complementary approaches, such as metagenomics, can characterize the lung microbiota with improved taxonomic resolution and provide an understanding of how lung bacteria affect clinical outcomes.

## 5. Conclusions

This pilot study demonstrated unique microbial community characteristics and immune cell changes in CLAD lungs compared to lung tissue at the time of transplantation. It is not yet clear whether these specific bacterial species are an evolution of a previous infection or are related to immunosuppressive treatment or CLAD. Nonetheless, the lung microbiome may be an important and understudied risk factor for lung allograft dysfunction. Further studies with a larger sample of patients are needed to determine whether changes in immune cell infiltration are associated with changes in the microbiome and how this affects CLAD.

## Figures and Tables

**Table 1:** Clinical variables of the patients.

Variables	P1665	P1760	P1763	P1961	Total (*n* = 4)
Age, years	36	53	61	68	57 [40.3–66.3]
Sex	F	F	M	M	M 2 (50)
BMI, kg/m^2^	26.2	22.5	19.0	25.1	23.8 [19.8–25.9]
Comorbidity					
Diabetes	−	+	+	+	3 (75)
Hypertension	−	−	+	−	1 (25)
Chronic kidney disease	−	−	+	+	2 (50)
Indication for first lung transplant					
	PPH	IPF	IPF	IPF	IPF 3 (75)
					PPH 1 (25)
Induction immunosuppression					
Basiliximab	+	+	+	+	4 (100)
Maintenance immunosuppression					
Calcineurin inhibitor					
Tacrolimus	+	+	+	+	4 (100)
Antiproliferative immunosuppression					
Mycophenolate	+		+	+	3 (75)
Azathioprine		+			1 (25)
Primary graft dysfunction immediately after transplant	−	−	−	−	0
Acute rejection	−	−	−	−	0
History of respiratory viral infection	+	−	−	+	2 (50)
History of respiratory bacterial infection	+	+	+	+	4 (100)
Previous microorganisms	*Pseudomonas aeruginosa*	*Acinetobacter baumannii*	*Escherichia coli*	*Klebsiella pneumoniae*	
History of CMV pneumonitis	−	−	−	−	0
Azithromycin for CLAD treatment	+	+	+	+	4 (100)
CLAD free survival, days	859	549	838	255	693.5 [328.5–853.8]

Data presented as median [interquartile range] or number (%). F; female, M; male, BMI; body mass index, +; existence, −; nonexistence, IPF; idiopathic pulmonary fibrosis, PPH; primary pulmonary hypertension, CLAD; chronic lung allograft dysfunction, CMV; cytomegalovirus.

## Data Availability

All data generated or analyzed during this study are included in this published article and its Supplementary Information Files. Raw data has been successfully registered with the BioProject database (BioProject ID: PRJNA879950).

## Supplementary Materials

The following supporting information can be downloaded at: https://www.mdpi.com/article/10.3390/microorganisms12020287/s1, Method S1: Standard tissue conservation technique; Method S2: DNA extraction, library construction, and sequencing; Method S3: RNA extraction, library construction, and sequencing; Figure S1: Overview of the data analysis workflow using Kraken 2; Table S1: Summary of the read counts for all patients. References [37,38,39,40] are cited in the supplementary materials.

## Abbreviations

CLAD	Chronic lung allograft dysfunction
ISHLT	International Society for Heart and Lung Transplantation
WGS	Whole genome sequencing
FPKM	Fragments per kilobase of transcript per million mapped reads
TIICs	Tumor-infiltrating immune cells
