# Supplementary material for: Unique Changes in the Lung Microbiome following the Development of Chronic Lung Allograft Dysfunction
Source: Microorganisms. 2024 Jan 29;12(2):287. doi: 10.3390/microorganisms12020287 (PMC10893466; doi:10.3390/microorganisms12020287)
Supplement: Supplementary file 1 [file microorganisms-12-00287-s001.zip › microorganisms-2830116-supplementary.pdf]

Supplementary method S1. Standard tissue conservation technique

Diseased human lungs and resected oversized lungs were collected in the PNUYH biobank with informed consent from patients with end-stage lung disease undergoing transplantation.

Using sterilized tweezers and a knife, slice the lung into thin slices, wash it several times with cold, sterile PBS, and place it in a sterilized cryotube. The cut lung pieces were immediately frozen in an ultra-low temperature freezer at -150 degrees and then moved and stored in a nitrogen tank, and some lung tissues were made into paraffin blocks.

Supplementary method S2. DNA extraction, library construction, and sequencing

The integrity of the genomic DNA was checked using agarose gel electrophoresis. gDNA was quantified using Quant-IT PicoGreen (Invitrogen, Thermo Fisher Scientific, Waltham, MA, USA). The sequencing libraries were prepared using the TruSeq DNA Nano Library Prep Kit from Illumina, Inc. (San Diego, CA, USA), following the manufacturer's instructions. Initially, 100 ng of genomic DNA was fragmented using adaptive-focused acoustic technology from Covaris LLC. (Woburn, MA, USA). The fragmented DNA was then subjected to end-repair, resulting in 5'-phosphorylated, blunt-ended double-stranded DNA molecules. Size selection of the DNA fragments was performed using a bead-based technique. The resulting fragments underwent the addition of a single 'A' base and ligation of TruSeq indexing adapters. The purified libraries were quantified using quantitative polymerase chain reaction (qPCR) following the qPCR Quantification Protocol Guide (KAPA Library Quantification Kits for Illumina Sequencing platforms). The libraries were further qualified using the Agilent Technologies 2200 TapeStation (Agilent Technologies, Santa Clara, CA, USA). Paired-end sequencing with a read length of 2x150 bp was carried out using the Macrogen platform in Seoul, Korea, and the NovaSeq platform from Illumina, Inc.

Supplementary method S3. RNA extraction, library construction, and sequencing

The total RNA was initially fragmented into smaller pieces using divalent cations at elevated temperatures. The resulting fragmented RNA was then used as a template for synthesizing first-strand cDNA using random primers. This was followed by the synthesis of second-strand cDNA. Subsequently, these cDNA fragments underwent an end repair process to ensure blunt ends, followed by adding a single 'A' base to the 3' ends of the fragments. Finally, adapters were ligated to the repaired and A-tailed cDNA fragments. To capture the human exonic region, we used the Agilent SureSelect XT Human All Exon V6+UTRs Kit, according to the standard Agilent SureSelect Target Enrichment protocol (Agilent Technologies). After the captured library was washed, it underwent a second round of PCR amplification. The final purified product was then quantified using Kapa Library Quantification kits specifically designed for the Illumina Sequencing platform (Illumina, Inc.), following the guidelines provided in the qPCR Quantification Protocol Guide (#KK4854, Kapa Biosystems Inc., Wilmington, MA, USA). The quality of the library was assessed using TapeStation D1000 ScreenTape (#5067-5582, Agilent Technologies). Indexed libraries were then sequenced using the Illumina NovaSeq system (Illumina, Inc.), and paired-end (2×100 bp) sequencing was performed using Macrogen (Seoul, Republic of Korea). The quality of individual sequences was evaluated using FastQC software [37] after adapter trimming with Trimmomatic (version 0.39) [38]. The generated reads were mapped against the genomic DNA reference (hg38) using HISAT2 [39]. Following the alignment of reads, the relative transcript abundance was determined using StringTie software [40]. This software calculates the abundance of each transcript in terms of fragments per kilobase of transcript per million mapped reads (FPKM).

References

37. Andrews S. FastQC: a quality control tool for high throughput sequence data. Available online at: http://www.bioinformatics.babraham.ac.uk/projects/fastqc accessed by 29 January 2024.
38. Bolger, A.M., M. Lohse, and B. Usadel, Trimmomatic: a flexible trimmer for Illumina sequence data. Bioinformatics, 2014. **30**(15): p. 2114-2120.
39. Kim, D., B. Langmead, and S.L. Salzberg, HISAT: a fast spliced aligner with low memory requirements. Nature Methods, 2015. **12**(4): p. 357-360.
40. Shumate, A., et al., Improved transcriptome assembly using a hybrid of long and short reads with StringTie. PLOS Computational Biology, 2022. **18**(6): p. e1009730.

Supplementary figure S1. Overview of the data analysis workflow using Kraken 2.

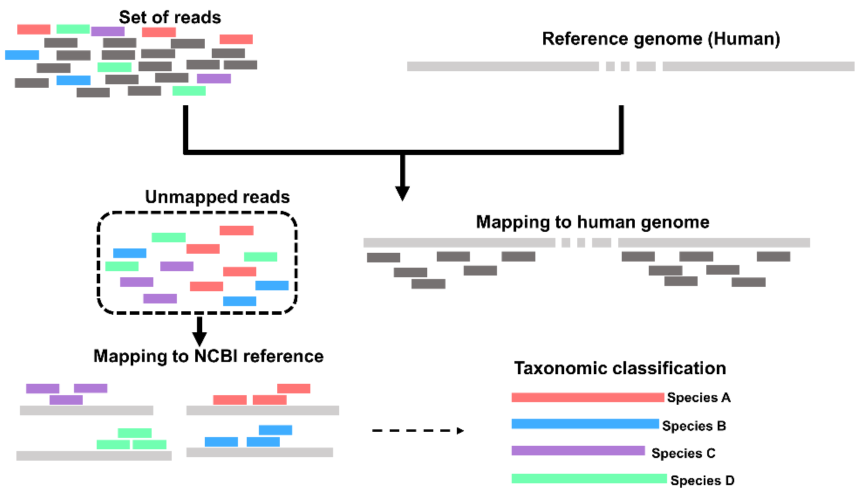

Sequencing reads that could not be aligned with known human reference genomes were mapped against a comprehensive database of known bacterial, archaeal, and viral microbial genomes using the ultrafast Kraken2 algorithm. The specific microbial database used in this analysis was the miniKraken DB_8GB.

Supplementary Table S1. Summary of the read counts for all patients.

	Total read	Microbiome read	Read assigned to microbiota (%)	Assigned Species
S1665_at transplantation	55441580	441315	0.796	91
S1760_ at transplantation	60183750	273234	0.454	50
S1763_ at transplantation	58207220	189756	0.326	94
S1961_ at transplantation	66120650	15208	0.023	16
S1665_CLAD	54073642	102740	0.19	71
S1760_CLAD	77769073	412176	0.53	184
S1763_CLAD	60236682	132521	0.22	63
S1961_CLAD	66460800	322335	0.485	51
